# Relation of baseline neutrophil-to-lymphocyte ratio to survival and toxicity in head and neck cancer patients treated with (chemo-) radiation

**DOI:** 10.1186/s13014-018-1159-y

**Published:** 2018-11-06

**Authors:** Beat Bojaxhiu, Arnoud J. Templeton, Olgun Elicin, Mohamed Shelan, Kathrin Zaugg, Marc Walser, Roland Giger, Daniel M. Aebersold, Alan Dal Pra

**Affiliations:** 1 Department of Radiation Oncology, Inselspital, Bern University Hospital and University of Bern, Freiburgstrasse, 3010 Bern, Switzerland; 2 Department of Medical Oncology, St. Claraspital Basel and Faculty of Medicine, University of Basel, Basel, Switzerland; 3 Department of Otorhinolaryngology, Head and Neck Surgery, Inselspital, Bern University Hospital, Basel, Switzerland; 4 Department of Radiation Oncology, Stadtspital Triemli, Zürich, Switzerland; 5 Center for Proton Therapy, Paul Scherrer Institute, Villigen, Switzerland

**Keywords:** Head and neck, Squamous cell carcinoma, Inflammation, Neutrophil-to-lymphocyte ratio, Platelet-to-lymphocyte ratio, Toxicity

## Abstract

**Background:**

A high neutrophil-to-lymphocyte ratio (NLR) is a marker of systemic inflammation and together with the platelet-to-lymphocyte ratio (PLR) is associated with worse outcomes in several solid tumors. We investigated the prognostic value of NLR and PLR in patients with head and neck squamous cell carcinoma (HNSCC) treated with primary or adjuvant (chemo)radiotherapy ((C)RT).

**Methods:**

A retrospective chart review of consecutive patients with HNSCC was performed. Neutrophil-to-lymphocyte ratio and PLR were computed using complete blood counts (CBCs) performed within 10 days before treatment start. The prognostic role of NLR and PLR was evaluated with univariable and multivariable Cox regression analyses adjusting for disease-specific prognostic factors. NLR and PLR were assessed as log-transformed continuous variables (log NLR and log PLR). Endpoints of interest were overall survival (OS), locoregional recurrence-free survival (LRFS), distant recurrence-free survival (DRFS), and acute toxicity.

**Results:**

We analyzed 186 patients treated from 2007 to 2010. Primary sites were oropharynx (45%), oral cavity (28%), hypopharynx (14%), and larynx (13%). Median follow-up was 49 months. Higher NLR was associated with OS (adjusted HR per 1 unit higher log NLR = 1.81 (1.16–2.81), *p* = 0.012), whereas no association could be shown with LRFS (HR = 1.49 (0,83-2,68), *p* = 0.182), DRFS (HR = 1.38 (0.65–3.22), *p* = 0.4), or acute toxicity grade ≥ 2. PLR was not associated with outcome, nor with toxicity.

**Conclusion:**

Our data suggest that in HNSCC patients treated with primary or adjuvant (C)RT, NLR is an independent predictor of mortality, but not disease-specific outcomes or toxicity. Neutrophil-to-lymphocyte ratio is a readily available biomarker that could improve pre-treatment prognostication and may be used for risk-stratification.

## Background

Risk stratification of patients diagnosed with head and neck squamous cell carcinoma (HNSCC) poses an important challenge [1]. Currently, some of the widely used factors are smoking and human papillomavirus (HPV) status, age, performance status, and tumor stage. Nomograms based on baseline characteristics can enhance prognostic prediction [2].

Inflammation is a hallmark of cancer [3], which is shown to play an important role in tumor development and progression [4–6]. An elevation of circulating neutrophil count is thought to be the result of tumor cells releasing cytokines, which stimulate the bone marrow to produce neutrophils [7–9]. Cytokines released by neutrophils also promote angiogenesis leading to tumor growth and metastasis [10–15]. There is an increasing interest in the use of hematological parameters as prognostic factors in malignancies. Neutrophil, lymphocyte, and platelet counts, either as individual values or in relation to each other, could be associated with the cancer prognosis [16, 17]. The neutrophil-to-lymphocyte ratio (NLR) is an emerging marker of host inflammation, which reflects the relation between circulating neutrophil and lymphocyte counts. It can be easily calculated from routine complete blood counts (CBCs) with differentiation. The independent prognostic value of NLR has been shown for a variety of solid malignancies [17–20]. In addition to NLR, the platelet-to-lymphocyte ratio (PLR) has also been shown to be a potential prognostic factor [2, 19]. Several studies involving HNSCC have shown an association between inflammation and worse prognosis [21–27]. However, information about the possible value of pretreatment NLR or PLR on toxicity is limited [18–29].

In this study, we retrospectively evaluated the prognostic impact of pretreatment NLR and PLR on oncological outcomes and toxicity in HNSCC patients treated with primary or adjuvant curative-intended (chemo-) radiotherapy ((C)RT). We hypothesized that elevated NLR and/or PLR are associated with detrimental survival; we also explored NRL and PLR associations with acute treatment-related toxicity since it has prognostic value in primary and adjuvant (C)RT for HNSCC [30, 31].

## Methods

### Patient selection

Medical records of HNSCC patients consecutively treated with primary or adjuvant curative-intent intensity-modulated radiation therapy with or without concomitant systemic therapy between January 2007 and December 2010 at the Department of Radiation Oncology, Inselspital, Bern University Hospital were retrospectively analyzed. Patients diagnosed with oral cavity (OCC), oropharynx (OC), hypopharynx (HC) and laryngeal cancers (LC) were included in the analysis. History of another malignancy within 5 years of diagnosis, prior radiation to the head and neck, non-squamous cell carcinoma histology, distant metastases, lack of differentiated CBC within 10 days before oncologic surgery or RT start, and early abortion of RT were defined as exclusion criteria. This study was approved by the local ethics committee (289/2014).

### Treatment and follow-up

The standard treatment was based on institutional policies following the multidisciplinary tumor board decision as previously published [32, 33]. All cases were presented at the weekly institutional interdisciplinary head-and-neck tumor board. After completion of staging examinations and final TNM staging (AJCC), selection of treatment modalities and treatment sequencing were defined. The standard treatment in OCC was to perform surgery followed by adjuvant radiotherapy (RT) [30, 32], while in OC, HC and LC the joint recommendations of the multidisciplinary meeting was primary RT [31, 33]. Case-based decisions were made concerning the use of concomitant systemic therapy and up-front neck dissection. The delivery of radiotherapy, the definition of clinical target volume (CTV) and planned target volume (PTV) followed departmental guidelines [32, 33] based on international recommendations [34–36]. All treatment plans were contoured and calculated using Eclipse treatment planning system (Varian Medical Systems, Palo Alto, CA). The standard concomitant therapy consisted of cisplatin 100 mg/m2 day 1 in three-week intervals for all patients. In few cases of induction chemotherapy, cisplatin, docetaxel, and 5-fluorouracil were used. Patients not deemed medically fit for cisplatin chemotherapy because of pre-existing co-morbidities were evaluated for weekly treatment with monoclonal antibody cetuximab [37] or carboplatin three weekly. Pre-treatment CBC with differential values was used to calculate NLR and PLR.

Potential causes of changes in the CBC (e.g. infection, steroid use) were identified, and patients were excluded from the analysis. Patients were regularly followed, and toxicities were graded according to the National Cancer Institute (NCI) Common Terminology Criteria for Adverse Events (CTCAE) version 4.03 (https://evs.nci.nih.gov/ftp1/CTCAE/CTCAE_4.03/CTCAE_4.03_2010-06-14_QuickReference_5x7.pdf).

### Statistical analysis

NLR was calculated by dividing absolute neutrophil count by absolute lymphocyte count measured in peripheral blood. PLR was calculated by dividing absolute thrombocyte count by absolute lymphocyte count. Due to its non-normal distribution, NLR and PLR were log_e_-transformed to obtain symmetric distributions and then analyzed as continuous variables. Frequencies and percentages are reported for categorical variables, medians with range or interquartile range for continuous variables. The primary endpoint of the study was overall survival (OS), and the secondary endpoints were locoregional relapse-free survival (LRFS) and distant recurrence-free survival (DRFS). Time-to-event was calculated for OS, LRFS, and DRFS from the start of RT to death (OS), locoregional relapse (LRFS), and distant recurrence (DRFS), respectively, with censoring of patients without such events at last follow up. Median times to event were estimated using the Kaplan Meier method. The prognostic value of NLR and PLR, and other variables (i.e. age, gender, smoking status, Karnofsky Performance Status (KPS), UICC stage, tumor grade, hemoglobin level) were assessed by univariable Cox regression analysis. Subsequently, multivariable analysis with forward elimination was planned with inclusion of all variables with a *p*-value < 0.05 in the univariable analysis. The association of NLR and PLR with acute and late toxicities (i.e. pain, dermatitis, mucositis, dysphagia, xerostomia) was examined using logistic regression. Analyses were carried out using SPSS version 23 (IBM Corp., Chicago, IL). The threshold for statistical significance was set at *p* < 0.05, and no correction for multiple testing was performed.

## Results

### Patients

One hundred and eighty-six patients were included in the study. Patients’ and disease characteristics are presented in Table 1. The majority of patients were male and in good performance status (KPS ≥ 70). The primary tumor was located in the oral cavity or oropharynx in approximately 75% of the cases, and more than half of all patients had UICC stage IVA or IVB disease. Median NLR and PLR were 3.28 and 189, respectively. There was a statistically significant correlation between NLR and PLR (Spearman’s rho = 0.65, *p* < 0.001). Baseline NLR and PLR were not associated with gender, smoking status, site of the primary tumor, stage of disease or tumor grade.

**Table 1:** Patients’ and disease characteristics

Age	
median (range), years	61 (41–88)
≤ 60, *N* (%)	86 (46)
> 60 to ≤70, *N* (%)	64 (34)
> 70 to ≤80, *N* (%)	27 (15)
> 80, *N (%)*	9 (5)
Gender, *N* (%)	
female	40 (22)
male	146 (79)
Smoking status	
never smoker	17 (6)
previous smoker	33 (31)
current smoker	58 (54)
missing	108
High risk alcohol consumption	
No	49 (46)
Yes	54 (51)
in the past	4 (4)
missing	79
Karnofsky Performance Status	
median (range)	90 (50–100)
> 70, *N* (%)	160 (86)
≤ 70, *N* (%)	26 (14)
Oncological resection of primary tumor	
yes	56 (30)
no	130 (70)
Induction chemotherapy	
yes	15 (8)
no	171 (92
Concomitant systemic therapy	
no	38 (20)
cisplatin or carboplatin	125 (67)
cetuximab	23 (12)
Site of primary tumor, *N* (%)	
oral cavity	52 (28)
oropharynx	83 (45)
hypopharynx	27 (15)
larynx	24 (13)
UICC stage, *N* (%)	
I	5 (3)
II	11 (6)
III	44 (24)
IV	126 (68)
Tumor grade, *N* (%)	
G1	1 (1)
G2	113 (61)
G3	72 (39)
Hemoglobin (g/dL)	
median (IQR)	13.3 (12.0–14.4)
missing	12
Neutrophil-to-lymphocyte ratio	
median (IQR)	3.28 (2.15–4.70)
missing	20
Platelet-to-lymphocyte ratio	
median (IQR)	189 (136–254)
missing	20

*IQR* inter-quartile range, *UICC* Union for International Cancer Control

### Overall survival

**Fig. 1:**
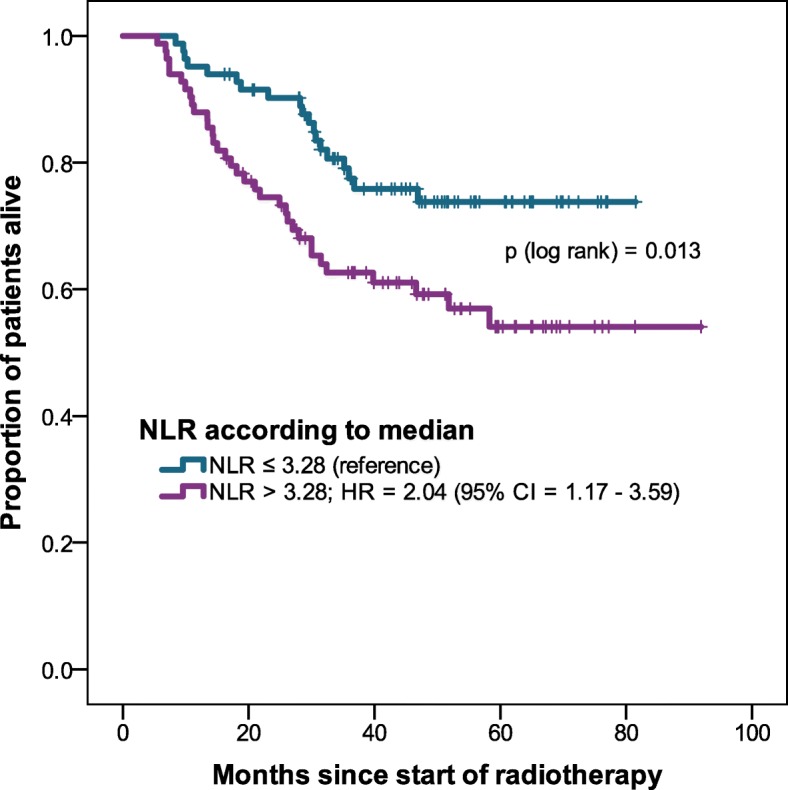
Overall survival of NLR higher than median vs. equal or lower than median

**Fig. 2:**
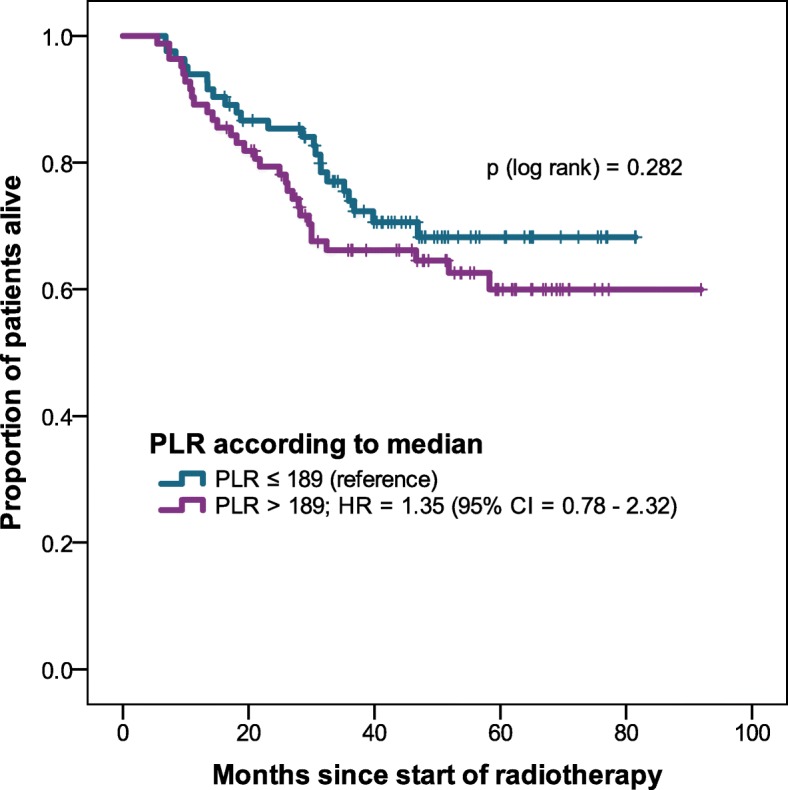
Overall survival of PLR higher than median vs. equal or lower than median

At a median follow-up time of 40 months, 60 patients (32%) died; median OS was not reached. Higher NLR was associated with lower OS (Table 2). When dividing the population into two groups according to the median NLR, there was a significant OS difference between the groups (Fig. 1). For PLR there was a non-significant association between higher PLR and lower OS (Fig. 2). On univariable analysis log_e_ NLR was associated with OS. Also, older age, worse Karnofsky Performance Status (KPS ≤ 70), and UICC stage IV were associated with lower OS. Performance status, UICC stage IV and log_e_ NLR remained of prognostic value in multivariable analysis (Table 2).

**Table 2:** Univariable and multivariable Cox regression analysis of overall survival

		univariable analysis		multivariable analysis	
		HR (95% CI)	*P*	HR (95% CI)	*P*
Age	per 10 years older	1.32 (1.03–1.69)	0.026*		
Gender	male (vs. female)	1.17 (0.61–2.25)	0.639		
Smoking status	never smoker (vs. current/past)	0.66 (0.20–2.19)	0.492		
Karnofsky Performance Status	per 10 higher	0.76 (0.62–0.92)	0.005*	0.76 (0.62–0.98)	0.030*
UICC stage	IVA-B (vs. I-III)	1.87 (1.01–3.47)	0.045*		
Tumor grade	G3 (vs. G1-G2)	0.91 (0.54–1.54)	0.731		
Hemoglobin	per 1 g/dL higher	0.89 (0.77–1.04)	0.143		
log NLR	per 1 log NLR higher	1.81 (1.16–2.81)	0.009*	1.58 (1.01–2.47)	0.043*
log PLR	per 1 log PLR higher	1.62 (0.99–2.63)	0.054		

*CI* confidence interval, *G* tumor grade, *HR* hazard ratio, *log NLR* natural logarithm of neutrophil-to-lymphocyte ratio, *log PLR* natural logarithm of platelet-to-lymphocyte ratio, *UICC* Union for International Cancer Control; *statistically significant

### Recurrence

**Fig. 3:**
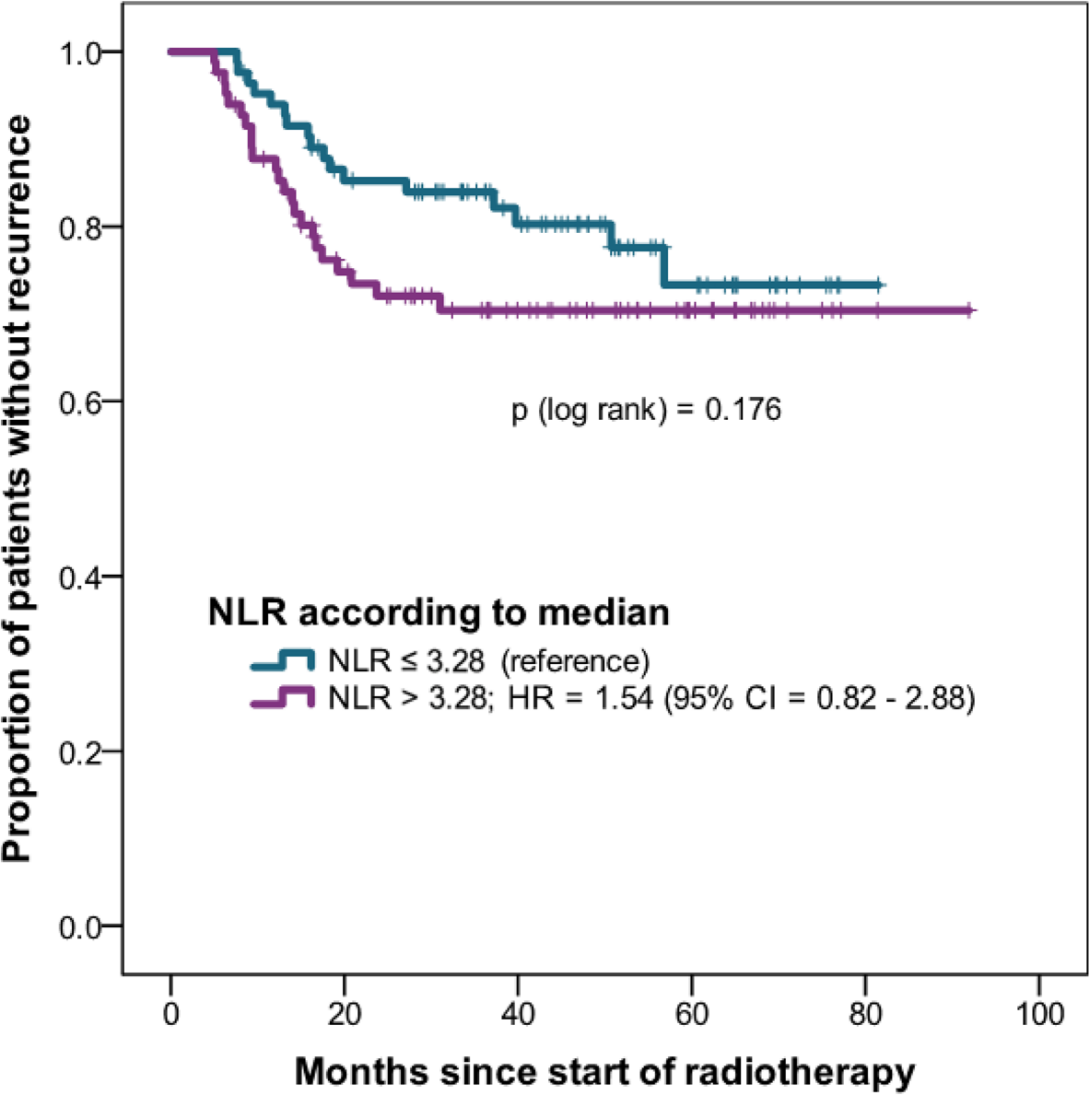
Recurrence-free survival of NLR higher than median vs. equal or lower than median

Of the variables tested, only UICC stage IV was associated with increased loco-regional, distant, and any recurrence rate, whereas no association was found for all other variables tested (Table 3). Consequently, no multivariable analyses were conducted. In patients with high NLR, recurrences occurred earlier, but the correlation was not statistically significant (Fig. 3).

**Table 3:** Univariable Cox regression analysis of recurrence

		Univariable analysis	
		HR (95% CI)	*P*
Loco-regional recurrence (38 events)			
Age	per 10 years older	1.08 (0.80–1.48)	0.607
Gender	male vs. female	1.21 (0.53–2.75)	0.648
Smoking status	never smoker (vs. current/past)	1.68 (0.34–8.31)	0.526
Karnofsky Performance Status	KPS over 70	0.55 (0.23–1.35)	0.191
UICC stage	IVA-B (vs. I-III)	3.43 (1.34–8.78)	0.010*
Tumor grade	G3 (vs. G1-G2)	0.78 (0.40–1.53)	0.477
Hemoglobin	per 1 g/dL higher	0.92 (0.76–1.13)	0.424
log NLR	per 1 log NLR higher	1.49 (0.83–2.68)	0.182
log PLR	per 1 log PLR higher	1.65 (0.88–3.10)	0.117
Distant recurrence (20 events)			
Age	per 10 years older	0.77 (0.49–1.22)	0.272
Gender	male	2.48 (0.57–10.7)	0.224
Smoking status	never smoker (vs. current/past)	0.04 (0.00–22.86)	0.314
Karnofsky Performance Status	KPS over 70	2.53 (0.34–18.94)	0.367
UICC stage	IV (vs. I-III)	9.91 (1.33–74.03)	0.025*
Tumor grade	G3 (vs. lower)	1.53 (0.64–3.68)	0.342
Hemoglobin	per 1 g/dL higher	1.11 (0.84–1.46)	0.472
log NLR	per 1 log NLR higher	1.38 (0.65–2.91)	0.400
log PLR	per 1 log PLR higher	1.44 (0.65–3.22)	0.371
Any recurrence (46 events)			
Age	per 10 years older	1.04 (0.78–1.28)	0.779
Gender	male	1.30 (0.61–2.79)	0.501
Smoking status	never smoker (vs. current/past)	0.60 (014–2.63)	0.501
Karnofsky Performance Status	KPS over 70	0.74 (0.33–1.65)	0.457
Localization	larynx or hypopharynx (vs. other)	1.24 (0.66–2.33)	0.497
UICC stage	IV (vs. I-III)	3.49 (1.48–8.24)	0.004*
Tumor grade	G3 (vs. G1-G2)	0.96 (0.53–1.74)	0.891
Hemoglobin	per 1 g/dL higher	0.95 (0.79–1.14)	0.948
log NLR	per 1 log NLR higher	1.49 (0.88–2.53)	0.134
log PLR	per 1 log PLR higher	1.55 (0.88–2.74)	0.128

*CI* confidence interval, *G* tumor grade, *HR* hazard ratio, *log NLR* natural logarithm of neutrophil-to-lymphocyte ratio, *log PLR* natural logarithm of platelet-to-lymphocyte ratio, *UICC* Union for International Cancer Control; *statistically significant

### Toxicity

Rates and grades of the most common acute toxicities are summarized in Table 4. There was no correlation between baseline NLR or PLR and the grade of toxicity (data not shown).

**Table 4:** Selected toxicities of 183 patients (toxicities of 3 patients missing)

	G1	G2	G3	G4
Symptoms prior to radiotherapy				
Pain	52 (28)	30 (16)	2 (1)	0
Dysphagia	52 (28)	32 (17)	11 (6)	0
Acute toxicities				
Pain	42 (23)	91 (49)	45 (24)	1 (1)
Dermatitis	44 (24)	117 (63)	22 (12)	0
Mucositis	31 (17)	110 (59)	40 (22)	0
Dysphagia	23 (12)	80 (43)	70 (38)	1 (1)
Xerostomia	63 (34)	8 (4)	0	0

Grades according to Common Terminology Criteria for Adverse Events (CTCAE) v4.03

## Discussion

NLR is the object of numerous previously published studies. Not only in oncology but also in other disciplines, blood counts reflecting the complexity of the immune system can be easily obtained at low costs, which may impact daily clinical practice. About 15–20% of all cancer deaths worldwide seem to be associated with underlying infections and inflammatory reactions [38]. Many triggers of chronic inflammation increase the risk of developing cancer. These triggers, for example, include microbial infections such as Helicobacter pylori (associated with stomach cancer), inflammatory bowel disease (associated with bowel cancer) and prostatitis (associated with prostate cancer) [38]. Despite conflicting studies, treatment with non-steroidal anti-inflammatory agents has been associated with reduced cancer incidence and mortality [38–41]. Increased NLR is associated with poorer outcomes in many solid tumors, be it early or advanced stage cancer [17]. An early decrease in NLR may be associated with more favorable outcomes and higher response rates [42], whereas an increase in NLR in the first weeks of treatment had the opposite effect [42].

In this study with a relatively large cohort of HNSCC patients treated with (C)RT with curative intention, an elevated NLR at baseline was associated with a shorter OS but not with disease recurrence or toxicities. Our findings of a negative prognostic role of NLR are in accordance with other studies [26, 43] that have investigated NLR in HNSCC. In contrast to our results, Rassouli et al. [44] have demonstrated a statistically significant impact of PLR on OS. Worth to note, such associations were observed at various cut-offs in different studies. They have also shown that an increased NLR was not only associated with decreased OS but with higher recurrence rates too [44]; which was not shown in our cohort and another study from the United Kingdom [45].

Along with the increased NLR in malignant disease, a possible explanation for a lower OS could also be a cause of death not attributable to cancer, but other co-morbidities such as a cardiac cause where it could also be shown that an increased NLR is predictive for cardiac mortality [46]. It is also known that smokers have a “smoker’s leukocytosis” [37, 38, 47, 48]. In our cohort, most patients are at least ex-smokers (80%), and at least one third continued smoking during and after radiation. Therefore, it is possible that the patients with a smoker’s leukocytosis have died earlier from smoking-related comorbidities [49].

Several limitations to our study should be considered. First, this was a retrospective analysis with possible selection bias and confounding variables. We included 16 patients (9%) with early-stage disease and 15 (8%) patients who had neoadjuvant chemotherapy, which may have introduced some heterogeneity to our cohort. Second, we were unable to capture data on HPV status systematically. Studies have shown an important interaction between HPV status, immunomodulation and clinical outcome [50]. Therefore, there might be different results in HPV-associated and unassociated tumors [51]. Since this is a retrospective study, there might be unknown causes of CBC changes that have not been identified. Beside patient and tumor-specific factors which may influence the complex cascades of the immune system, it must also be noted that despite clinical benefit, the dichotomization or grouping of continuous variables in statistical analysis is accompanied by a loss of the statistical power. To account for this, NLR and PLR were analyzed as (log-transformed) continuous variables. Lastly, an overestimation of statistical significance due to multiple testing is possible. Although these results should be validated in other cohorts, we reproduced some of the previously reported studies [26, 43] on the interface of systemic inflammatory pathways and OS. Therefore, we provide data on surrogate values for inflammation as predictors of clinical outcomes; however, a causal relationship and its impact on tumor aggressiveness or tumor microenvironment warrants further investigation.

## Conclusion

Our data suggest that in HNSCC patients treated with primary or adjuvant (C)RT, NLR is an independent predictor of OS. NLR is a readily available biomarker that could improve pre-treatment risk stratification.

## Abbreviations

(C)RT: (Chemo)radiotherapy
CBCs: Complete blood counts
CI: Confidence interval
DRFS: Distant recurrence-free survival
G: Tumor grade
HC: Hypopharyngeal cancer
HNSCC: Head and neck squamous cell carcinoma
HR: Hazard ratio
IQR: Inter-quartile range
LC: Laryngeal cancer
log NLR: Natural logarithm of neutrophil-to-lymphocyte ratio
log PLR: Natural logarithm of platelet-to-lymphocyte ratio
LRFS: Locoregional recurrence-free survival
NLR: Neutrophil-to-lymphocyte ratio
OC: Oropharyngeal cancer
OCC: Oral cavity cancer
OS: Overall survival
PLR: Platelet-to-lymphocyte ratio
RT: Radiotherapy
UICC: Union for International Cancer Control
